# Electroanalytical overview: recent advances in the sensing of arsenic using screen-printed electrochemical platforms

**DOI:** 10.1039/d5en00708a

**Published:** 2026-01-02

**Authors:** Jessica L. Pimlott, Dale A. C. Brownson, Edward P. Randviir, Eric M. Brack, Craig E. Banks, Samuel J. Rowley-Neale

**Affiliations:** a Electrochemistry Group, Manchester Metropolitan University Dalton Building, Chester Street Manchester M1 5GD UK s.rowley-neale@mmu.ac.uk; b U.S. Army Combat Capabilities Development Command (DEVCOM)—Soldier Center 10 General Greene Avenue Natick Massachusetts 01760 USA

## Abstract

Access to clean drinking water remains a critical global health issue, with over 2 billion people lacking access to safely managed water. Among the contaminants of concern, heavy metals (particularly arsenic) pose significant risks due to their persistence and toxicity. Long-term exposure to arsenic (As^3+^), especially at concentrations exceeding the World Health Organisation's (WHO) guideline of 10 μg L^−1^, has been linked to severe health conditions, including cancer, dermatological issues, and cognitive impairments. Traditional laboratory-based approaches offer high sensitivity and selectivity but are expensive, time-consuming, and require skilled personnel, making them impractical for in-the-field use. In recent years, electrochemical methods, particularly using screen-printed electrodes (SPEs), have emerged as a promising alternative for As^3+^ detection, the most toxic form. SPEs offer a compact, cost-effective, and portable solution, enabling real-time, on-site monitoring of arsenic levels in water. This review systematically explores recent advancements in SPEs for the detection of As^3+^, with a focus on electrode modifications aimed at enhancing sensitivity and selectivity. We highlight techniques involving the integration of precious metals, biosensors, and carbon-based materials, all of which contribute to improved (lower) detection limits and wide sensing ranges. Practical applications in environmental monitoring (particularly in remote or resource-limited settings) are also discussed, offering a scalable and efficient solution for arsenic detection; SPEs have the potential to revolutionise water quality assessment and supporting global public health initiatives.

## Abstract 2

SPEs towards the detection of arsenic with a focus on electrode modifications to enhance sensitivity and selectivity.

## Graphical abstract

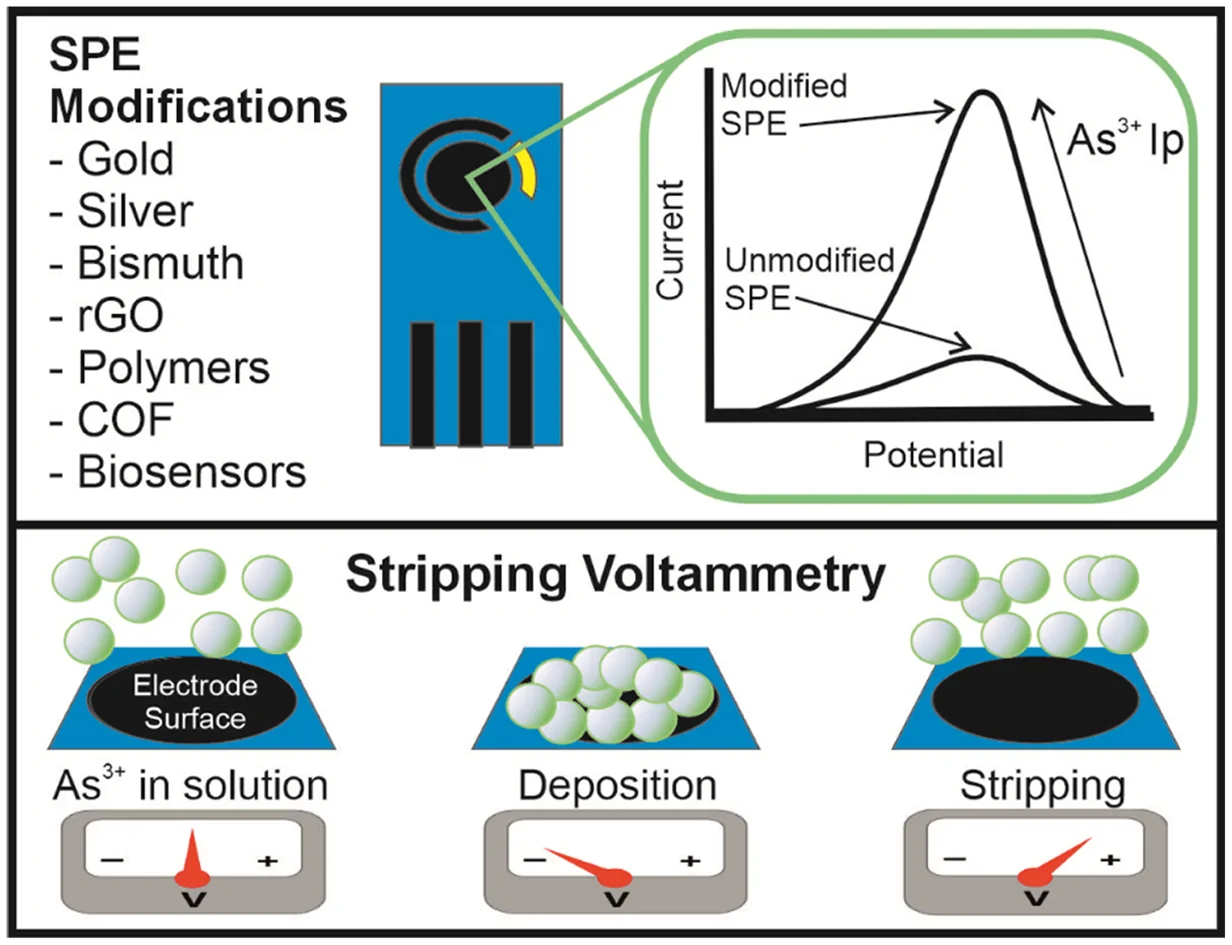

**Environmental significance**

This review addresses a critical environmental and public health challenge: the detection of arsenic (As^3+^) in drinking water, which continues to threaten over 2 billion people worldwide. By evaluating recent advancements in nanomaterial-modified screen-printed electrodes (SPEs), the work highlights how nano-enabled sensing technologies can provide portable, low-cost, and sensitive solutions for real-time water quality monitoring. The integration of nanostructured materials; such as carbon nanomaterials, metal nanoparticles, and biosensors, not only enhances analytical performance but also facilitates deployment in remote and resource-limited regions. This review underscores the transformative potential of nanotechnology in decentralised water testing, supporting global efforts toward safe water access and sustainable environmental monitoring.

## 1. Introduction

Access to clean drinking water is an essential requirement for human health, yet the World Health Organisation (WHO) estimates that over 2 billion people lack access to safely managed drinking water.^1^ Among the numerous threats to water quality, heavy metal (HM) contaminants are of particular concern due to their persistence in the environment and associated toxicity.^2–4^ These HM contaminants often infiltrate groundwater and drinking water supplies, primarily because of anthropogenic activities related to industrial processes and agricultural practices, a summary of which is presented in Table 1. In response to the serious health risks posed by HMs, the WHO and Environmental Protection Agency (EPA) outline strict exposure limits to mitigate the detrimental effects on human health.^5^

**Table 1:** Summary of common heavy metal environmental contaminants, their origins, and regulatory limits for drinking water, according to the World Health Organisation (WHO) and the Environmental Protection Agency (EPA)

Metal contaminant	Natural sources	Anthropogenic sources	WHO limit,^a^ μg L^−1^	EPA limit,^a^ μg L^−1^	Ref.
Antimony	Mineral deposits	Metal alloying, lead-acid batteries, fire retardants	20	6	6
Arsenic	Volcanic activity, mineral desorption and dissolution	Metal smelting, mining, wood preservatives	10^b^	10	7
Cadmium	Volcanic activity, erosion, sulphide ores of Zn and Pb	Tobacco smoking, mining, Ni–Cd batteries, municipal waste incineration	3	5	8
Chromium	Chromite ore	Stainless steel, welding, cement works	50	100	9
Copper	Naturally occurring in soils	Numerous industrial and agricultural processes	2000	1300	10
Lead	Soils and clays	Vehicle exhausts, paints, plumbing and welding	10^b^	15	11
Mercury	Bioaccumulation in edible aquatic species	Dental amalgam work, artisanal gold mining	6	2	12
Nickel	Rocks and minerals, release from wildfires and windblown sands	Mining and smelting, cigarettes, fossil fuel combustion	70	—	13
Selenium	Sulphide ores of Cu, Pb, Ni, Au, and Ag	Glass production, copper, ceramic and plastic pigments	40	50	14

a Based on μg L^−1^ (ppb) in drinking water.

b Changing to 5 μg L^−1^. WHO: World Health Organisation. EPA: Environmental Protection Agency.

Among the range of HMs, arsenic poses a significant public health challenge.^15^ Arsenic is a naturally occurring contaminant, with seawater, unpolluted groundwater, and freshwater usually having an arsenic concentration of 2 μg L^−1^, 1–10 μg L^−1^, and 0.15–0.45 μg L^−1^, respectively.^16–18^ Whilst arsenic can be found naturally in a variety of oxidation states, it is the arsenite (3+) oxidation state that is particularly toxic and therefore of greatest concern to human health.^16^

**Fig. 1:**
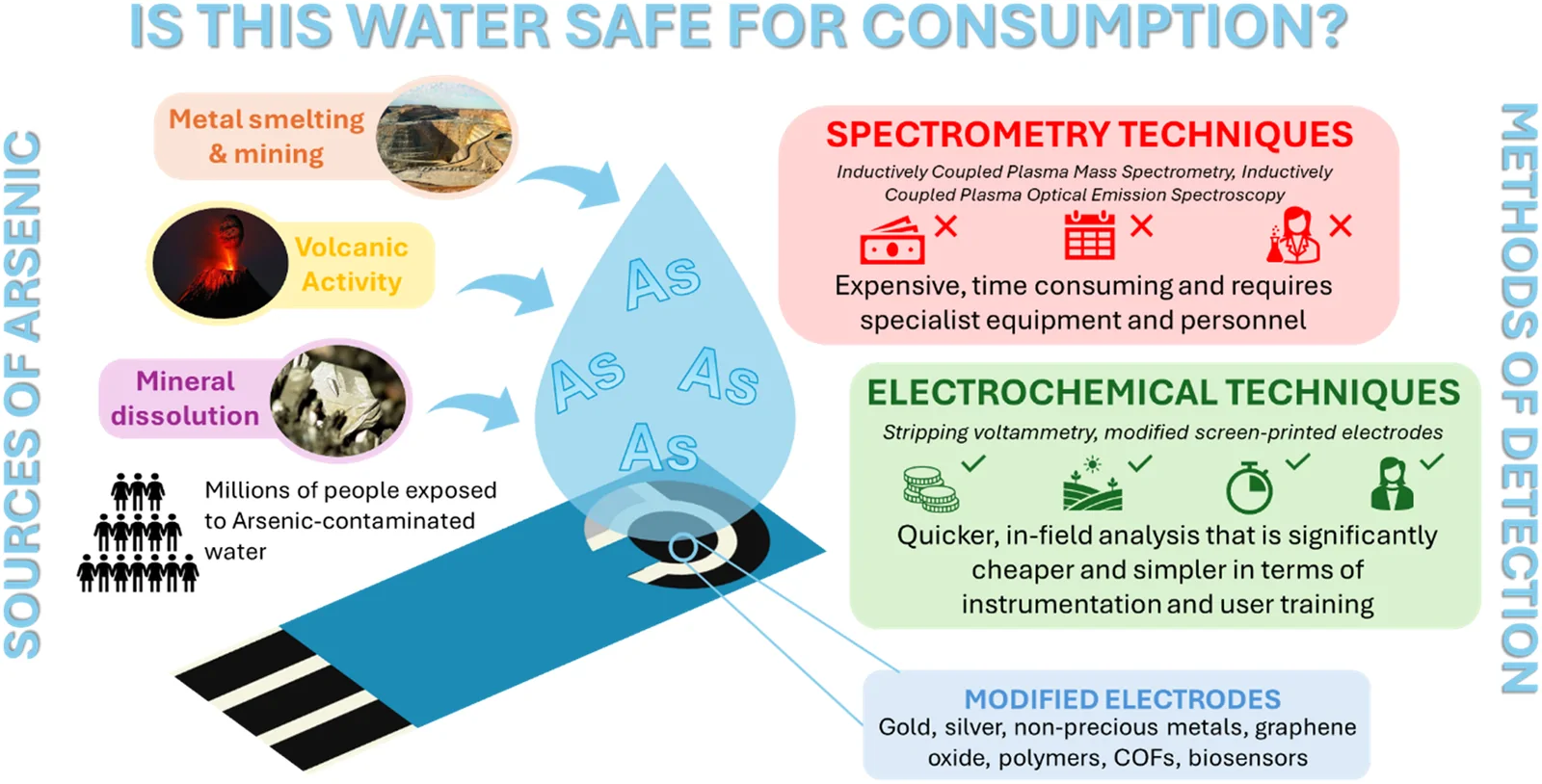
Overview of arsenic contamination sources and subsequent arsenic detection methods.

Anthropogenic activities have been known to cause significant levels of arsenic contamination within drinking water, for example, individuals in the Ballia district of India (2013) were shown to be drinking water that had an arsenic concentration of *ca.* 370 μg L^−1^, resulting in severe health implications for the effected individuals.^19^ Podgorski and Berg identified that between 94–220 million people worldwide are potentially exposed to groundwater sources that contain arsenic concentrations higher than the WHO exposure limit.^20^ Chronic arsenic exposure is associated with severe health concerns, such as dermatological legions, cognitive and behavioural impairments, and heightened cancer risks.^21–23^ Arsenic typically enters the human body *via* ingestion, skin absorption, and inhalation, after which it sequesters within a variety of organs, such as the skin, liver, kidneys, and lungs.^24,25^ Arsenic poisoning is known as arsenicosis, which is usually very difficult to diagnose as its symptoms are often nonspecific and can be attributed to numerous other diseases.^26^ These health impacts underscore the urgent need for effective monitoring and management strategies to ensure the safety of drinking water supplies, and the health of the global population (Fig. 1).

The detection and quantification of arsenic is necessary if the detrimental health consequences are to be avoided. In drinking water samples this is typically performed with advanced laboratory-based analytical techniques, such as inductively coupled plasma optical emission spectroscopy (ICP-OES) and inductively coupled plasma mass spectroscopy (ICP-MS).^13,27–29^ While these techniques are renowned for their high sensitivity, accuracy, and capability to detect concentrations at sub-part per trillion (ppt, ng L^−1^) levels, they also present significant challenges. These challenges include high operational costs, extensive time requirements for sample analysis, and the need for specially trained personnel to operate the instrument.^30^ This limits their implementation to regions that have the necessary resources and manpower, meaning that often geographical locations that are the most at risk of acute levels of arsenic within their drinking water are the areas that cannot sufficiently test for it. As a result, there is a pressing need for alternative methods that can provide timely and cost-effective solutions for arsenic detection.

One such solution is colorimetric methods for arsenic quantification. Colorimetric test kits for arsenic detection remain popular due to their simplicity, portability, and low cost, making them suitable for rapid screening in remote or resource-limited settings.^31^ These assays allow on-site visual assessment of contamination without specialised instrumentation. However, their reliability is limited by subjective colour interpretation, user-dependent variability, and poor precision near regulatory thresholds. Many kits also require toxic reagents, long reaction times, and provide only semi-quantitative results, often underestimating arsenic in real water samples. Even recent nanomaterial-based colorimetric sensors improve sensitivity yet still suffer from variable stability, matrix effects, and challenges in quantifying trace As^3+^ and As^5+^ in complex environmental waters.^32^ In contrast to the aforementioned techniques, electrochemical detection procedures offer a practical alternative with shorter analysis times, simple methodologies, and significantly lower equipment costs.^33^ To address the need for portable and efficient in-field arsenic detection, the development of screen-printed electrodes (SPEs) presents a promising solution. SPEs facilitate the integration of the three-electrode setup into a compact and disposable configuration that can be easily paired with a handheld potentiostat. This combination makes SPEs ideally suited to rapid, on-site water quality measurements. The advantages of SPEs extend beyond their portability; they also facilitate more frequent and extensive testing, particularly in remote or resource-limited environments where traditional laboratory-based methods are impractical.^34,35^

In this review, a systematic examination of recent advancements in electrochemical methods for arsenic detection is presented, with a particular focus on the transition from traditional laboratory-based techniques to portable, field-ready solutions facilitated by SPEs. Emphasis is placed on innovations in electrode design, surface modifications to enhance sensitivity and selectivity, and the integration of novel detection strategies for the quantification of arsenic. This includes the use of nanomaterials, such as gold nanoparticles, carbon nanotubes, and conducting polymers, which have been shown to enhance the electrochemical response of the electrodes. By highlighting the advancements and practical implications of SPE technology, this review provides insights into how these innovative approaches can contribute to global efforts in ensuring access to safe drinking water, thereby safeguarding public health, and promoting sustainable development. The ability to rapidly assess water quality *in situ* can empower communities, inform regulatory decisions, and facilitate more effective water management strategies, ultimately contributing to the reduction of arsenic-related health risks.

## 2. Electrochemistry of arsenic

The electrochemical behaviour of arsenic is critical for the development of efficient and reliable sensing platforms, particularly when using SPEs for detection in infield scenarios. Understanding the speciation and redox chemistry of arsenic under various conditions is essential for optimisation of sensor design and associated electrochemical detection methods. In the environment, arsenic is typically found in one of four oxidation states; arsenate (5+), arsenite (3+), arsenic (0) and arsine (3−). Given that the toxicity, mobility, and reactivity of arsenic is strongly influenced by its oxidation state, monitoring these transformations *in situ* is crucial for both environmental assessment and remediation efforts.

**Fig. 2:**
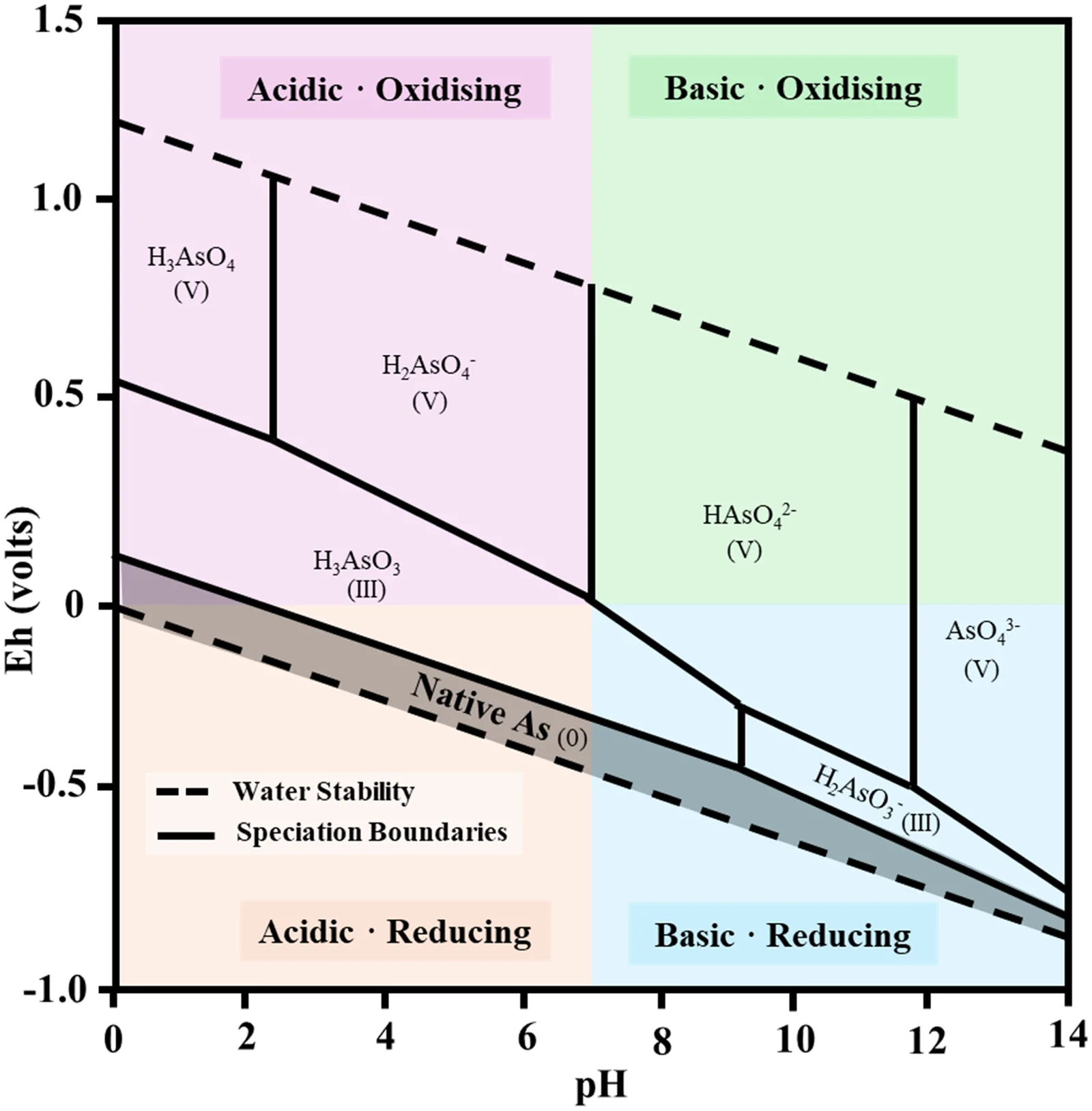
Pourbaix diagram of arsenic speciation at 25 °C and 1 bar. Coloured regions indicate acidic/basic and oxidizing/reducing environments. Dashed lines show water stability and solid lines represent As speciation boundaries. Oxidation states are indicated. Overall, the diagram display that As(v) species dominate under oxidising conditions, As(iii) under reducing conditions, and native As^0^ is stable at low Eh near the water-stability limit. Figure reproduced from ref. 37 with permission from Springer Nature,^37^ copyright 2025.

The speciation of arsenic in aqueous solutions is highly dependent on pH and redox potential, as depicted in the Pourbaix diagram of Fig. 2. This plot of potential (Eh) against the pH of the solution delineates the thermodynamically stable phases of arsenic, including arsenite (As^3+^) and arsenate (As^5+^). Under acidic conditions at lower pH values, trivalent arsenite species such as H_3_AsO_3_ and H_2_AsO_3_^−^ dominate, particularly in mildly reducing environments. As the pH increases into the alkaline region, pentavalent arsenate species like H_2_AsO_4_^−^ and HAsO_4_^2−^ become more stable. Additionally, the redox potential plays a crucial role in these transitions. In oxidizing environments, arsenate species are the predominant form due to the higher oxidation state of As(v), while in reducing conditions, such as anoxic waters or sediments, arsenite is more likely to be present. For example, the dominant forms of arsenic in groundwater are a combination of both As(iii) and As(v), whereas in well water as much as 80% of total arsenic is comprised of the more toxic As(iii) form.^36^

The line boundaries on the Pourbaix diagram represent the equilibrium lines, where the conversion between species occurs.

The electrochemical reduction of arsenic(iii) is shown by the following process:As(iii)(aq) + e^−^(m) → As(ii)(aq) rdsAs(ii)(aq) + e^−^(m) → As(i)(aq) equilibriumAs(i)(aq) + e^−^(m) → As(0)(ads) equilibriumwhich can be observed using gold nanoparticles, which are identical to that at a gold single crystal electrode.^38^

The corresponding stripping wave is due to the electrochemical stripping of arsenic metal to arsenic(iii) with the peak height (and of course the peak area) proportional to arsenic(iii) concentration, providing the corresponding analytical signal. Interestingly, the choice of electrolyte significantly impacts the oxidation potential. In arsenic detection, acidic electrolytes like HCl are commonly employed because chloride ions facilitate ionic conduction between the working electrode and the target As^3+^ species. This enhances the efficiency of the electrochemical detection by providing a stable ionic pathway.^36^

**Fig. 3:**
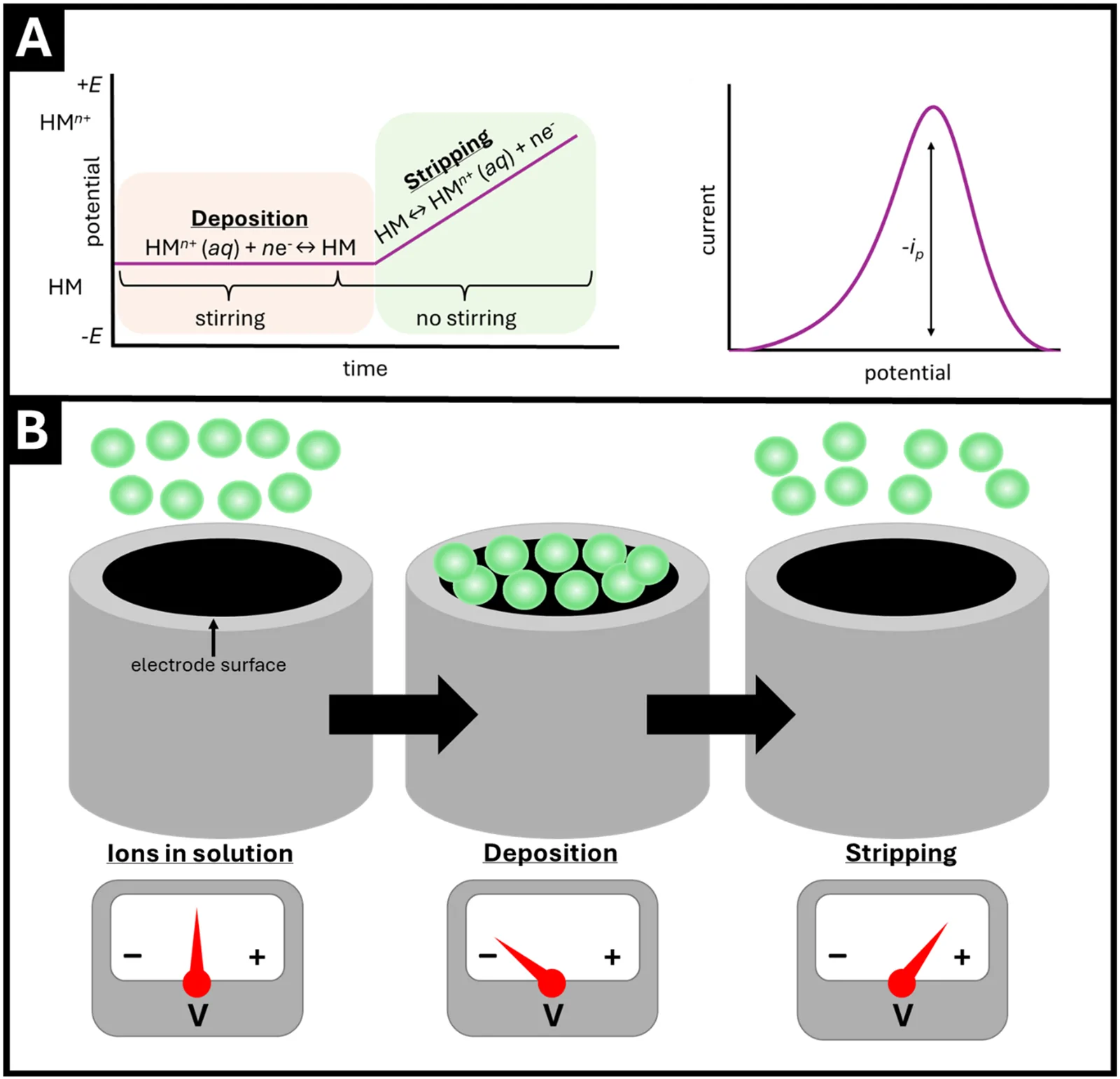
Schematic of the anodic stripping voltammetry (ASV) method. A) Displays the concentration of HM and subsequent oxidation at the electrode surface during the progression of the two-step deposition and stripping progression of the ASV method, and resulting spike in current proportional to analyte concentration. B) The behaviour of the analyte species at the electrode surface as the potential changed during the deposition and stripping steps.

One such electrochemical detection technique is anodic stripping voltammetry (ASV). ASV is frequently employed for arsenic detection due to its high sensitivity and suitability for in field and low concentration analysis. ASV operates through a two-step process – deposition and stripping (as seen in Fig. 3A). In the deposition phase, HM ions are reduced and deposited onto the electrode surface under a controlled negative potential (time, potential, and level of solution agitation can be adjusted here to optimise the method). During the subsequent stripping phase, a potential sweep in the positive direction oxidises the deposited HM, generating a measurable current that is directly proportional to its concentration. An overview of the electrochemical activity at the electrode surface is shown in Fig. 3B. Different analytes will oxidise at different potentials, allowing for simultaneous detection of multiple elements in a single electrochemical method.

In addition to the role of electrolyte composition and pH, the choice of electrode material is crucial in determining the efficiency and sensitivity of arsenic detection. Different electrode materials exhibit varying affinities for arsenic species, influencing both the kinetics and thermodynamics of the electrochemical reactions involved. For example, gold electrodes are commonly employed in the electrochemical detection of arsenic due to their high catalytic activity and affinity for arsenic species, particularly As^3+^. The surface properties of gold facilitate the adsorption of arsenite, enhancing electron transfer and lowering the overpotential required for oxidation to As^5+^.^39^ In contrast, carbon-based electrodes, such as glassy carbon or carbon paste electrodes, offer lower costs and exhibit good electrochemical stability, although they may require surface modifications, such as nanoparticle coatings or chemical functionalisation, to improve their sensitivity towards arsenic species. Recent developments in nanostructured electrodes, including materials like gold nanoparticles or modified graphene, have shown promise in further enhancing the detection limits and selectivity for arsenic by increasing surface area and facilitating more efficient electron transfer.

## 3. Screen-printed electrodes

Traditional solid carbon-based electrodes, such as those comprising glassy carbon (GC), do not effectively translate to in-field use as they are often relatively expensive, delicate yet bulky, and require cleaning in-between experiments. Screen-printed electrodes (SPEs) overcome the in-field limitations of traditional carbon-based electrodes with their cheap, disposable, and mass-producible nature.^34,35,40,41^ This review now considers their fabrication and application as sensing platforms towards arsenic.

Screen-printing techniques have been used for centuries, entailing the use of a mesh to transfer designs onto various substrates, including plastics, fabrics, ceramics, and organic surfaces. More recently, this technology has found a critical role in the field of electronics, benefiting from its highly reproducible and cost-effective nature. Screen-printing using automatic technology enables the rapid, large-scale manufacture of customised electrodes and electronic components, making it an ideal technique for applications in circuit printing, wearable electronics, and other flexible systems.^42,43^ High-precision automatic screen-printing facilitates the production of flexible electrodes that can be developed into advanced sensing devices, including SPEs, which form the foundation of many modern electrochemical detection platforms.^34,35^ SPEs are a versatile and efficient tool in the field of electrochemical sensing towards numerous applications, such as environmental/water monitoring, clinical diagnostics, and food safety.^44^ Their compact, disposable, and portable nature allows the integration of multiple electrodes into a single sensing platform, enhancing their convenience for in-field analysis.

**Fig. 4:**
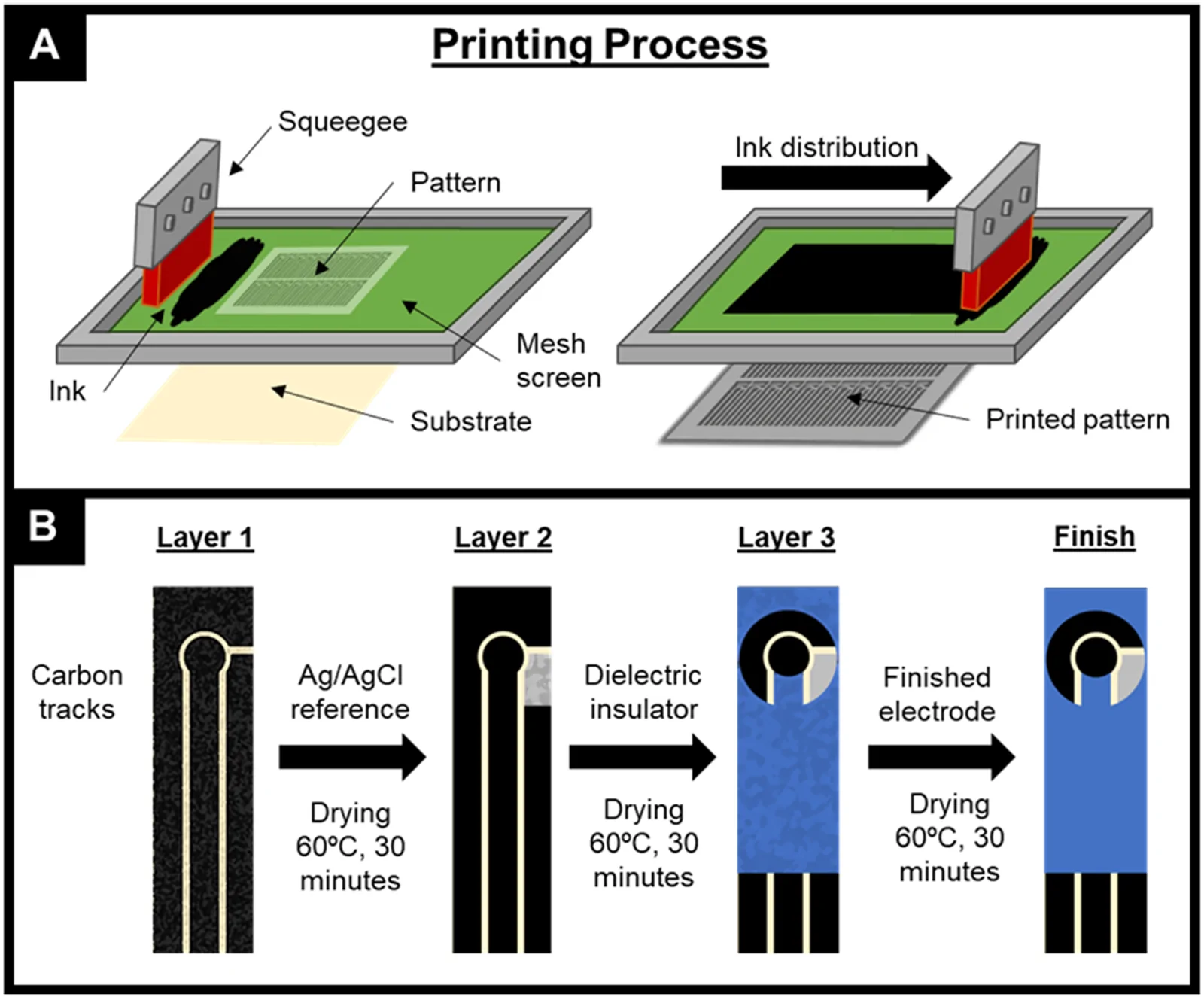
A schematic of the screen-printing process to create a standard screen-printed electrode, where A) shows the distribution of ink over a mesh screen to transfer an electrode pattern onto the chosen substrate. Each distinct layer is displayed in B), revealing the initial carbon track and counter electrode layer, the Ag/AgCl reference layer and the final dielectric layer with curing times proceeding each ink layer application.

Screen-printing entails the use of a mesh screen to transfer a pattern onto a chosen substrate using an ink. Commonly used substrates for SPEs include plastic, ceramic and paper.^34,35^Fig. 4A depicts the mesh screen with a pre-defined electrode pattern. During printing, an ink is guided over the mesh by a squeegee or ink blade, the ink is forced through the mesh, printing the electrode pattern onto the substrate below. In Fig. 4B the conductive tracks are printed from a carbon ink, but this may be replaced with a plethora of commercially available alternative inks. After a curing step, the time of which is unique to each ink type, the next layer is applied. A reference electrode, usually consisting of a Ag/AgCl ink, is then added, followed by a layer of insulating ink to define the electrode areas.

Due to the adaptability of the screen-printing process, SPEs can be fabricated with various geometries and compositions, and tailored to suit numerous applications. Common types of SPE include macroelectrode, microelectrodes, microarrays, flow-cell type electrodes, in addition to multiplex working electrode platforms. Microelectrodes, which feature a miniaturised version of the working electrode, offer high sensitivity and lower detection limits due to enhanced mass transport and reduced ohmic losses.^45^ These miniaturised systems are particularly useful in detecting trace analytes, making them invaluable for applications requiring high sensitivity, such as detecting trace levels of heavy metals or organic pollutants in environmental samples.^46^

SPEs have been successfully used to monitor contaminants such as lead, cadmium, and mercury in diverse water samples, including tap water, river water, and groundwater.^47–49^ The incorporation of modification strategies—such as the use of nanoparticles, conducting polymers, and other nanostructures—has further enhanced the sensitivity and selectivity of SPEs for heavy metal detection. These modifications make SPEs a powerful tool for monitoring toxic metal concentrations, which is critical for safeguarding public health and ensuring environmental protection.

It is clear that SPEs represent a versatile, scalable, and cost-effective solution for the electrochemical detection of heavy metals, particularly arsenic, in a variety of environmental and industrial settings. The detection of arsenic using SPEs has been significantly enhanced through various modification strategies aimed at improving sensitivity, selectivity, and detection limits, an overview of the current state-of-the-art modifications are presented in Table 2 for instance, SPEs modified with gold nanoparticles or metal oxides, have demonstrated increased affinity for arsenic species, particularly arsenite (As^3+^), due to their catalytic properties and high surface area.^38,50–53^ Furthermore, carbon-based SPEs, modified with graphene or carbon nanotubes, have also been employed to increase the surface conductivity and active surface area, leading to lower detection limits for arsenic in water samples. Incorporating conducting polymers, such as polyaniline or polypyrrole, onto SPEs has further improved the selectivity towards arsenic by promoting selective adsorption of arsenic species at the electrode surface. Moreover, SPEs can be integrated with advanced sensing technologies, such as microfluidics and flow-injection analysis, to enable real-time, on-site detection of arsenic with high precision and reproducibility. These platforms allow for continuous monitoring in contaminated water sources, ensuring rapid responses to fluctuating arsenic levels.

**Table 2:** Overview of modified SPE towards detection of arsenic in numerous lab-based and real-world samples

Modification	Method	Electrode	Electrolyte/sample	LOD, μg L^−1^	Linear range, μg L^−1^	Ref.
BiVO_4_ nanoflakes, polyaniline	DPASV	Carbon SPE	Acetate buffer	0.0072	0.01–300	54
Silica nanoparticles	LSASV	Carbon SPE	Groundwater	6.2	5–30	55
Silica nanoparticles/gold nanoparticles	LSASV	Carbon SPE	Water samples	5.6	10–100	50
Ultrathin quasi-hexagonal gold nanostructures	DPV	Carbon SPE	Tap water	0.038	0.075–30	51
Gold nanostars	DPV	Carbon SPE	Tap water	—	0–100	52
			River water			
Gold nanoparticles	LSASV	Carbon SPE	Canal water	0.4	19–75	38
Carbon nanotubes modified with gold nanoparticles	LSASV	Vibrating carbon SPE	1 M HCl	0.5	0.05–134	53
([Ru(bpy)_3_]^2+^–GO)	DPV	Carbon SPE	Citrate buffer	0.021/0.034	0.08–15	56
				As^3+^/As^5+^		
Benzotriazole–rGO	DPV	Carbon SPE	0.1 HCl	0.217	0.150–3.0	57
Gold–rGO	SWV	Carbon SPE	0.1 M PBS	1.07	0.00075–74.9	58
Platinum nanoparticles	CV	Carbon SPE	Tap water	5.68		59
Silver nanoseeds	DPASV	Carbon-fibre SPE	Tap water	0.6	1.9–25.1	60
Fe_3_O_4_–Au–ionic liquid	SWASV	Carbon SPE	Synthetic river water	2.4	0–50	61
Bipyridine functionalised COF	SWASV	Carbon SPE	River water	2.85	7.49–5993	62

Ongoing research into the functionalisation of SPEs with novel nanomaterials and molecular recognition elements will likely continue to improve the performance of these sensors, making them even more sensitive and selective for arsenic detection in complex environmental matrices. The most promising technologies/materials for SPE modification to enhance the electrochemical determination of As^3+^ are explored next.

## 4. Metal modifications

Metal-modified SPEs have been extensively investigated for arsenic detection due to their high catalytic activity, tuneable surface chemistry, and compatibility with miniaturised electrochemical platforms. Incorporating metallic nanostructures onto the working electrode surface can dramatically enhance electron-transfer kinetics, improving both detection sensitivity and selectivity. Precious metals such as gold, silver, and platinum are among the most widely employed owing to their superior conductivity and affinity toward arsenic species, particularly As^3+^. However, growing interest in sustainability and cost reduction has driven the exploration of non-precious alternatives, including bismuth, tungsten, and silica-based systems, which offer environmentally benign and economically viable sensing options. This section reviews recent advances in metal-modified SPEs, highlighting how different metallic components influence analytical performance, the mechanisms underlying arsenic detection, and the ongoing challenges related to surface stability, selectivity, and scalability.

### 4.1. Gold modifications

As evident in the section above, gold-based enhancements are particularly prominent in electroanalytical sensing due to their ability to significantly increase current outputs, thereby lowering the LOD for target analytes such as arsenic. Gold nanoparticles (AuNPs) are especially advantageous, offering high conductivity, a large surface area, and superior catalytic properties that facilitate improved electron transfer at the electrode surface.

#### 4.1.1. Gold Nano-particle synthesis

AuNPs are most commonly synthesized using the well-established Turkevich method, in which sodium citrate reduces tetrachloroauric acid (HAuCl_4_), yielding spherical nanoparticles in the nanometre size range.^63^ Variations of this method have been developed over the decades, notably the Turkevich–Frens method, which allows greater control over particle size and distribution by adjusting reagent concentrations and reaction conditions.^64^ This section explores the various approaches and advances in gold nanoparticle-modified SPEs, highlighting their impact on sensitivity, selectivity, and practicality in real-world arsenic sensing applications.

In terms of cost, the chloroauric acid precursors for AuNPs are relatively expensive, limiting large-scale production. Alternatively, Nascimento *et al.* obtained a gold precursor from the central processing units (CPUs) of obsolete computer systems.^65^ This source of gold produces nanoparticles *via* the Turkevich method, however, impurities such as silver and tin required complex methodologies to extract and isolate the gold. Similarly, work by Su-Gallegos *et al.* synthesised HAuCl_4_ from recovered gold coatings of processor board pins obtained from e-waste.^66^ The resulting HAuCl_4_ was then used to produce AuNPs following a Turkevich methodology.

#### 4.1.2. Gold Nano-particle electrode modification

The implementation of gold nanostructures as modifications for carbon SPEs and their subsequent utilisation towards detection is of growing interest to the field. There are numerous techniques implemented within the literature to modify electrodes with Au-NPs. The most common method of electrode modification is drop-casting, where an aliquot of the explored material (whilst in a liquid suspension) is dropped onto the surface of the electrode, usually using an automated micropipette. The electrode is then given time to dry, in which the suspending liquid evaporates, and the material (*i.e.* Au-NPs) remains on the surface of the electrode where it is electrically wired.^67^ Udayan *et al.*^51^ used this technique to prepare quasi-hexagonal AuNPs of between 15 and 18 nm in diameter. These were initially synthesized by boiling a 10 mM HAuCl_4_ solution with a 1% sodium citrate mix. After 60 seconds, a visual colour change indicated the formation of AuNPs. To achieve the final sensor platform, the quasi-hexagonal AuNPs were drop-casted onto a commercially available SPE with a 3 mm diameter working electrode. The sensor was tested in both a 0.1 M PBS and a spiked tap water sample. Furthermore, the authors found that the electrode achieved excellent reproducibility with a %RSD of 1.1% and an observed signal deviation of 4% when the same concentration was quantified seven days apart.^68^

#### 4.1.3. Interference from HM contaminants when using gold modifications

**Fig. 5:**
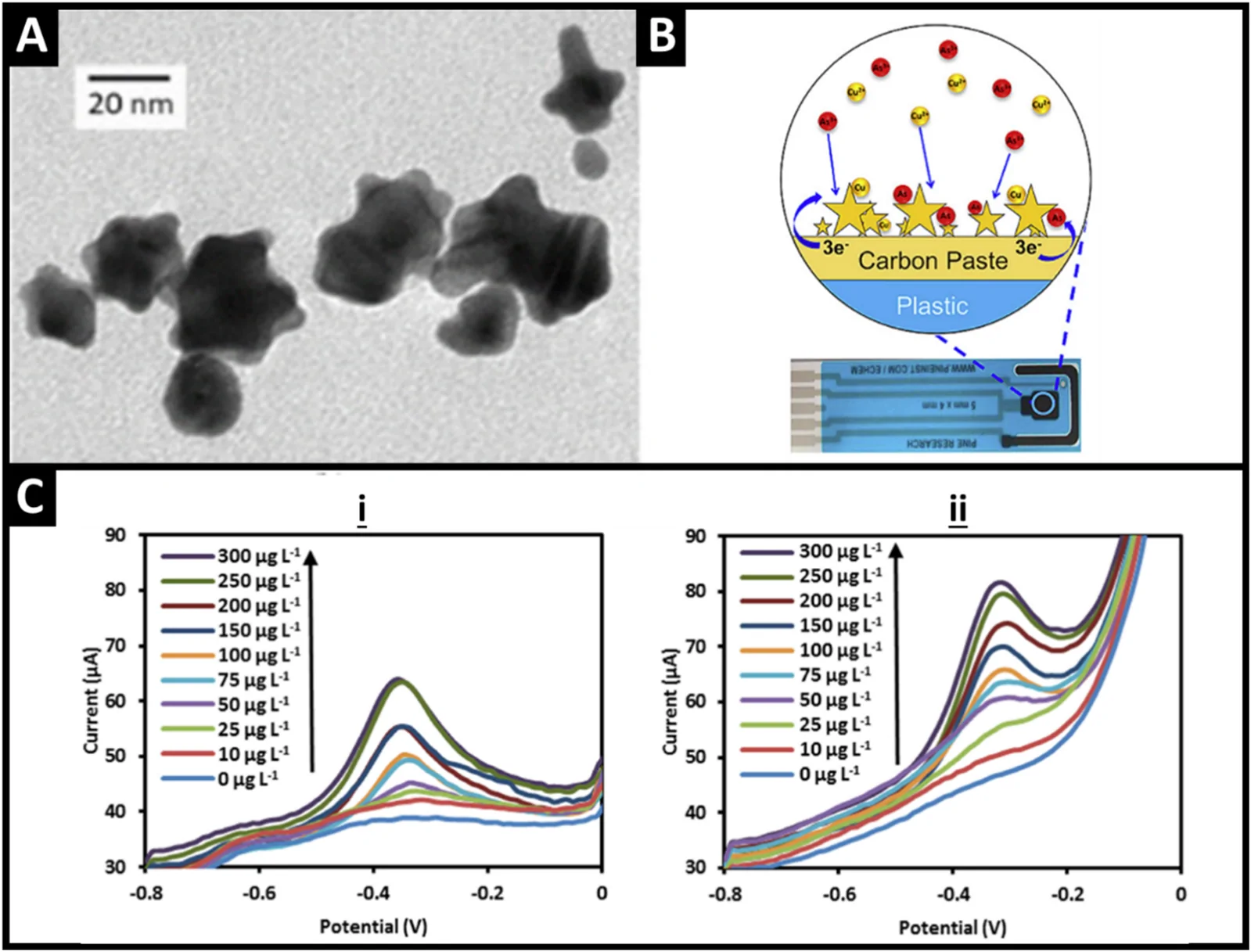
A) A transition electron microscopy (TEM) image showing the morphology of gold nanostar particles in the nanometre size range. B) Shows a depiction of the modified carbon screen-printed electrode for simultaneous quantification of arsenic and copper. C) Voltammograms for arsenic concentrations between 0 and 300 μg L^−1^ with i) no copper and ii) 1.3 mg L^−1^ of copper. Figure reproduced from ref. 69 with permission from Elsevier,^69^ copyright 2020.

When utilising gold modified electrodes towards arsenic detection, the interference of prominent contaminants, such as Cu^2+^, should be considered. Researchers have developed techniques to help deconvolute the obtained electrochemical signal outputs to allow for a more accurate determination of the HM's present within a tested sample. For example, Sullivan *et al.*^69^ report on the fabrication of gold nanostars supported on a carbon SPE for co-detection of As^3+^ and Cu^2+^ in alkaline Britton Robinson buffer, real river, and tap water samples. The authors prepared the gold nanostars by combining a heated HAuCl_4_ solution with a solution of HEPES buffer and 10 M NaOH in a 1 : 100 ratio. The solution was stirred then left undisturbed for 20 minutes at room temperature to allow the formation of AuNP prior to refrigeration. The final morphology of the resulting gold nanostars was revealed using a transition electron microscope (TEM), as shown in Fig. 5A. The gold nanostars were then drop-casted onto a carbon SPE with a rectangular working electrode with an area of 20 mm^2^ as depicted in Fig. 5B. In the case of a spiked tap water sample, the LODs for Cu^2+^ and As^3+^ were 42.5 and 2.9 μL^−1^. The peaks obtained *via* a square wave anodic stripping voltammetry (SWASV) method displayed good separation of peaks for both species, allowing for efficient codetection. The voltammograms in Fig. 5C show comparable peak heights for As^3+^ concentrations first without Cu^2+^ and secondly in the presence of 1.3 mg L^−1^ Cu^2+^.

When tested in real-world samples of river water, the voltametric results agreed well with graphite furnace atomic absorption spectroscopy (GF-AAS) data, showing As^2+^ recoveries of 87% and 88%, respectively. However, voltametric measurements for tap water samples showed an average RSD of over 10% at an 83% recovery rate.

Similarly, Khairy and co-workers^38^ explored the detection of As^3+^ in the presence of Cu^2+^ in canal water samples. Citrate-capped AuNPs were immobilised onto a carbon SPE and used to detect As^3+^ concentrations in over the linear range of 19–75 μg L^−1^. To achieve this, the canal water samples were diluted to 10% with 1 M HCl to avoid interference from other environmental water constituents. To further expand the remit of gold-based As^3+^ sensors, Lu and coworkers^52^ investigated the feasibility of simultaneous detection of As^3+^, Cd^2+^ and Se^4+^ with LODs of 0.8, 1.6 and 1.6 μg L^−1^, respectively. The SWASV method achieved well-defined peaks for all three target species, the height of which were reduced by approximately 40% during simultaneous detection due to the formation of electrochemically inactive species such as arsenic triselenide, however, this did not affect the quantification ability. When compared to AAS, the average recovery rate for As^3+^ was 93% in river water samples. Clearly, employing AuNPs constituents of novel morphologies allow for benefits in both the catalytic surface enhancement over the underlying electrode, and improvements in mass transport regimes, giving rise to beneficial sensor performances.

A method of combining AuNP modifications with 1,6-hexanedithiol was reported by Etorki *et al.*,^70^ to detect the total arsenic concentration in a range of water and food samples. To fabricate the sensor, AuNPs were electrodeposited onto the electrode surface, by flooding the working electrode with a 6 mM HAuCl_4_ in 0.1 M HNO_3_ solution whilst applying a potential of −0.4 V. The electrode was then submerged in a 1,6-hexanedithiol solution for a minimum of 18 hours to promote the self-assembling of a 1,6-hexanedithiol monolayer. The resulting sensors were applied to the detection of As^3+^ in tea and coffee samples, achieving a LOD of 1.7 ng mL^−1^. The combination of SPEs modified with Au nanoparticles offer a promising in-field sensing platform they are however typically single use. This has the potential to create issues regarding water and disposal of the SPEs.^34^ However, Punrat and coworkers^71^ developed a sequential injection/ASV technique that enables the repeated use of a single SPE for detection purposes. Sequential injection techniques are popular flow-based systems often used as an automation route for routine analysis where high-throughput is desired. Using this method, a single Au-modified SPE could be used over 300 times whilst maintaining a %RSD of below 2.7%. However, this stability test was conducted under laboratory conditions with a solution of 3 mg L^−1^ As^3+^ in 1 M HCl, which is not necessarily representative of electrode stability towards real world water samples. When the technique and electrode were applied to speciation tests, good recovery efficiency was achieved for As^3+^ and As^5+^ at 99% and 97%, respectively.^71^

#### 4.1.4. Complex matrices

The majority of studies are performed in water samples, there is however a real-world requirement to detect arsenic in various matrices, such as in fruit juices and other beverages. Sullivan *et al.*^72^ evaluated the feasibility of quantification in apple juice. In many commercially available fruit juices ascorbic acid is added to enhance nutritional values, however, this can interfere with the voltametric detection of heavy metals. The carbon SPE in this work modified with AuNP was able to detect As^3+^ concentrations as low as 16.73 μg L^−1^ in apple juice samples buffered with a phosphate buffer. The authors noted that the As detection is not interfered with by the presence of ascorbic acid as there is no competition for As binding sites or formation of any As complexes.

Work by Gamboa *et al.*^53^ reports the detection of arsenic by adhering carbon nanotubes modified with AuNPs to the working electrode of a carbon SPE. By incorporating a vibrating motor to the SPE as opposed to the traditional magnetic stirrer used during the deposition stage of voltametric techniques, effects from external agitation are mitigated. The authors used a LSASV (linear sweep) method with a deposition time of 120 s to achieve a 0.5 μg L^−1^ LOD with a %RSD of 2.4% over 10 measurements in a 1.0 M HCl solution. Furthermore, with increasing concentrations of Cu^2+^ of up to 1.5 mg L^−1^, no interference with the As^3+^ peak was observed, showing a promising technique for analysis of samples with high Cu^2+^ concentrations. The field should aim to diversify the matrices utilised in studies to explore the applicability of electrochemical detection methods into more niche real-world applications.

### 4.2. Other precious metals

Alongside gold, other precious metal nanoparticles are used in sensing arsenic. Sanllorente-Méndez and coworkers,^59^ modified a carbon SPE with Pt nanoparticles to detect As^3+^ concentrations as low as 5.68 μg L^−1^ in spiked tap water samples. Once Pt nanoparticles were electrodeposited onto the working electrode of a carbon SPE, cyclic voltammetry was performed, revealing an additional peak alongside the typical As^3+^, which the authors attribute to the formation of PtOH. Commonly interfering species such as Zn^2+^, Se^4+^, Fe^3+^ and Ni^2+^ amongst others did not cause any interference on the As^3+^ oxidation peak, presenting a promising detection method for determination of As^3+^ in complex water samples.^59^ Expanding on this work, Melinte *et al.*^73^ created a nanocomposite SPE, containing both Pt and Au nanoparticles. The modification of the SPE involved the electropolymerisation of analine, followed by the electrochemical co-deposition of Pt and Au nanoparticles from their corresponding salts, H_2_PtCl_6_ and HauCl_4_ SPE, creating a hybrid bimetallic and polyanaline film on the working electrode surface. The total deposition time for this technique was 12 minutes. Using a SWASV method, the SPE achieved an LOD of 19.7 nM for As^3+^ with a linear range of 0–200 nM. The bimetallic SPE performed better than comparable SPEs with a single Au or Pt nanoparticle modification.

**Fig. 6:**
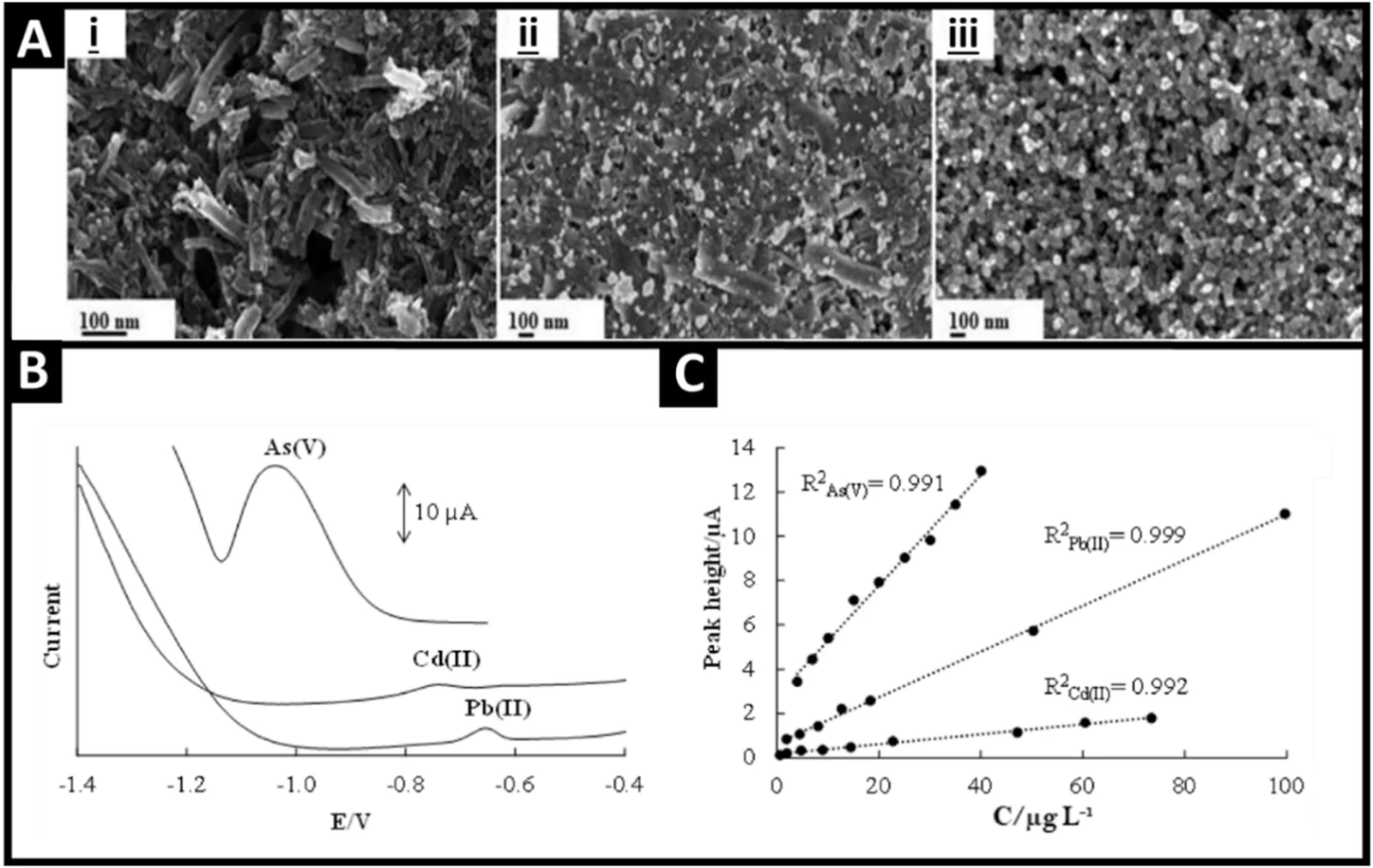
A) i) SEM images of unmodified carbon-nanofiber based SPEs and electrodes drop-cast with ii) silver nanoseeds and iii) silver nanoprisms. B) Differential pulse anodic stripping voltammograms pf As^5+^, Cd^2+^ and Pb^2+^ at concentrations of 25 μg L^−1^ and C) calibration plots at a deposition potential of −1.4 V in an acetate buffer at pH 4.5. Figure reproduced from ref. 74 with permission from MDPI,^74^ copyright 2021.

Silver has also been utilised as a modification for SPEs towards detection purposes. Torres-Rivero *et al.*^60^ compares the ability of carbon nanofiber SPEs (Fig. 6A) i) modified with either silver nanoprisms (Fig. 6A) ii) or silver nanoseeds (Fig. 6A) iii) to detect As^5+^ in water samples. To synthesise the silver nanoseeds, a silver nitrate solution was added continuously to a trisodium citrate and sodium borohydride mixture. To alter the nanoseed morphology to form nanoprisms, Milli-Q water and ascorbic acid was added alongside further silver nitrate additions. The resulting nanoseed and nanoprism solutions were then drop-casted onto carbon-fibre SPEs and allowed to dry. The electrodes were tested in spiked tap water samples for the determination of As^5+^, the results of which were validated against ICP-MS. Although both electrodes were capable of detecting As^5+^ at concentrations well below the WHO exposure limits, the SPE coated with silver nanoseeds exhibited the best electrochemical performance, with a LOQ of 0.6 μg L^−1^, compared to 1.9 μg L^−1^ for the silver nanoprisms. The LOD was 0.6 μg L^−1^ in the linear range of 1.9–25.1 μg L^−1^, presenting a suitable option for As^5+^ determination at low concentrations.^60^ To fully investigate the sensor's ability, the authors published further work, showing that the carbon nano-fibre silver nanoseed SPE could simultaneously detect Cd^2+^ and Pb^2+^ alongside As^5+^. Fig. 6B shows the three well-defined peaks for all three species with LODs for Pb, Cd and As at 3.3, 3.7 and 2.6 μg L^−1^, respectively. This indicates that the presence of interfering species negatively effects the LOD for As^5+^ from 0.6 μg L^−1^ for single species detection, to 2.6 μg L^−1^ for multi-analyte analysis.^74^

Other elements, such as ruthenium (Ru), have been used towards the detection of arsenic. Gupta *et al.*^75^ electrodeposited Ru nanoparticles onto a glassy carbon electrode, detecting As^3+^ at concentrations as low as 0.1 μg L^−1^. However, this work has not been transferred into a portable set up, such as a SPE, limiting the in-field use of this sensor.

### 4.3. Non-precious metals

Bismuth modifications are prevalent throughout sensing literature due to its ability to form eutectic alloys with ions of heavy metals such as Cd and Pb, enhancing analyte deposition on the electrode surface and consequently improving electrochemical responses.^76^ Although, Bi is rarely used in sensors for As, Durai *et al.*^54^ reported a BiVO_4_/polyaniline modification synthesised *via* a single-step hydrothermal synthesis. The resulting modified carbon SPE sensor achieved an impressively low calculated LOD of 7.2 ng L^−1^ As^3+^ in blood samples with visible current peaks down to 10 ng L^−1^ using a DPV method. Furthermore, the Bi-based sensor covered a linear range of 0.01–300 μg L^−1^, encompassing a range of global As exposure guideline limits. Although capable of detection in a range of electrolytes, degradation of the sensor occurred at pH values exceeding 4.5.^54^

In addition, Zhao *et al.*^61^ developed a nanocomposite SPE with two working electrodes for the simultaneous detection of Cd^2+^, Pb^2+^ and As^3+^. The first working electrode was modified with an Fe_3_O_4_–Au–ionic liquid nanocomposite for As^3+^ detection and the second with a (BiO)_2_CO_3_–rGO–Nafion nanocomposite for Cd^2+^ and Pb^2+^ detection. The resulting electrode was incorporated into a 3d-printed flow injection system to analyse simulated river water samples. Using a deposition time between 200 and 250 seconds, and a flow rate of 120 μL s^−1^, a 2.4 μg L^−1^ LOD was achieved for As^3+^ with a %RSD of 2.28%. The authors tested a range of non-target ions for interference, of which only Cu^2+^ interfered with As^3+^ detection at 100-fold increased concentrations. The results suggest a promising tool for automated detection of As^3+^ alongside other common HM contaminants, however, further validation in real world water samples is necessary. A further example of Fe_3_O_4_ as an SPE modification is seen in work by Gao *et al.*^77^ who utilised the developed platform to achieve a detection limit of 8 × 10^−4^ ppb and a sensitivity of 4.91 μA ppb^−1^.

A carbon SPE modified with tungsten disulfide nanosheets (WS_2_NSs) was able to detect roxarsone, a poultry feed additive, concentrations in the linear range of 0.05 to 489.3 μM. The sensor showed exceptional selectivity amongst interfering biomolecules such as nitrites, glucose, propyl gallate and various acids that naturally occur in the body.^78^

Silica nanoparticles were investigated towards electroanalytical assays. Ismail *et al.* report a silica nanoparticle modified SPE that achieves a LOD of 6.2 μg L^−1^ in groundwater samples, without the inclusion of precious metals. The silica modified SPE is comparable to a sensor modified with silica/Au nanoparticles that achieved detection down to a 5.6 μg L^−1^ limit. Although the use of silica nanoparticles is desirable from a sustainability point of view, inclusion of Au nanoparticles extends the achievable linear range from 5–30 μg L^−1^ to 10–100 μg L^−1^.^50,55^

SPEs modified with both precious and non-precious metals have shown considerable promise for arsenic detection, but each presents specific material and operational challenges. AuNP-modified SPEs provide high sensitivity toward As^3+^ yet suffer from surface fouling, metal–ion interference, and nanoparticle aggregation during cycling. These issues can be reduced by applying antifouling polymer coatings, embedding AuNPs within conductive carbon matrices, and functionalising surfaces with thiol or aptamer ligands for greater selectivity and stability. Silver-modified electrodes readily oxidise or undergo sulfidation processes, forming passivating layers that degrade sensitivity. Their durability can be improved through alloying with gold or palladium, protective graphene or chitosan coatings, and chelating additives to suppress interference. Platinum-based SPEs, though highly conductive, are costly and prone to chloride or sulphide poisoning and hydrogen evolution. These drawbacks may be alleviated *via* Pt–Ru or Pt–Ni alloying or controlled nanoparticle dispersion. Among non-precious metals, bismuth is beneficial from an environmental standpoint but limited film stability, while tungsten and silica suffer from oxide instability and poor conductivity. These can be addressed through bismuth-carbon composites, oxide doping, or nano-structuring of tungsten, and conductive filler incorporation in silica matrices to enhance charge transfer and robustness.

Future research should focus on the rational design of multi-metallic nanocomposite architectures that combine the catalytic efficiency of precious metals with the stability and sustainability of non-precious alternatives. Integrating these materials within 3D conductive carbon frameworks or porous polymer/carbon supports could enhance surface accessibility and long-term durability. Coupling such systems with microfluidic sample handling, machine-learning-assisted signal interpretation, and renewable or green synthesis routes would enable intelligent, low-cost, and field-deployable platforms for continuous arsenic monitoring, bridging the gap between laboratory precision and real-world environmental applications.

## 5. Carbon-based modifications

This section reviews recent literature highlighting the use of carbon-based material modified SPEs, exploring their synthesis methods, functionalization strategies, and electrochemical performance in arsenic detection across various environmental applications.

### 5.1. Graphene oxide modifications

**Fig. 7:**
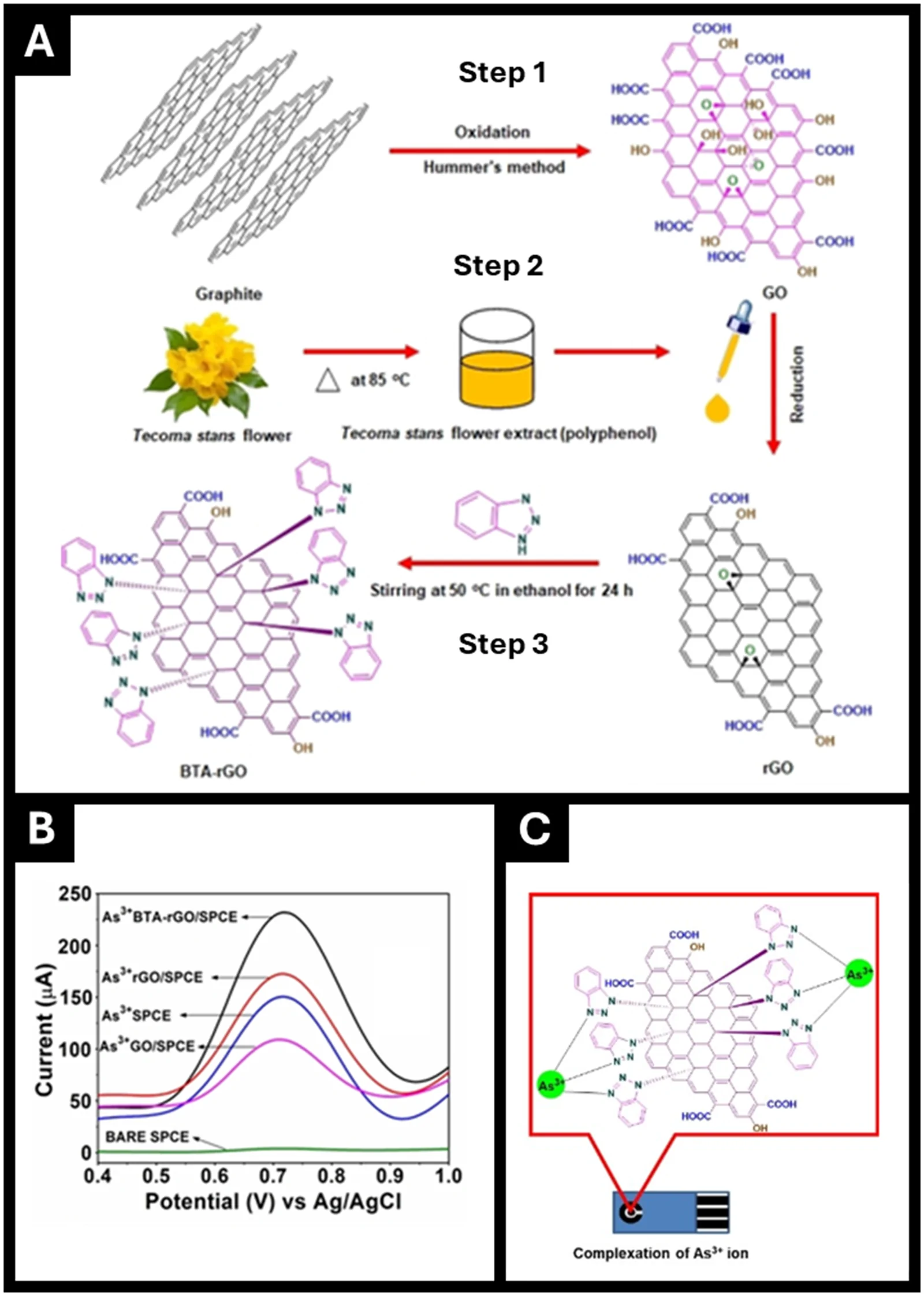
(A) Illustration of synthesis of BTA–RGO, (B) DPV current response of various modified electrodes in the presence of As^3+^ (0.02 μM) in HCl (0.1 M) solution and (C) proposed mechanism of arsenic detection. Figure adapted from ref. 57 with permission from Wiley,^57^ copyright 2022.

Chemical reduction of GO is the most common route for production of reduced graphene oxide (rGO), however, this typically necessitates toxic reducing agents. Work by Gunasekaran *et al.*^57^ investigated the use of a sustainable alternative, namely, a *Tecoma stans* flower extract, the overall process of which is shown in Fig. 7. Initially, graphite was oxidised into GO using a traditional Hummer's method. The resulting GO was then reduced using the *Tecoma stans* flower extract to achieve stabilised rGO nanosheets. To further enhance conductivity, benzotriazole was covalently bonded onto the rGO surfaces using a nucleophilic substitution (SN_2_) method to produce the benzotriazole–rGO shown in Fig. 7A. Finally, the benzotriazole–rGO was drop-casted onto the working electrode of a carbon SPE. Compared to an unmodified carbon SPE, the benzotriazole–rGO–SPE showed an increase in conductivity from 539 to 102 Ω. When compared electrochemically, the modified benzotriazole–rGO–SPE showed a significantly higher current signal than equivalent SPEs modified with just GO or rGO, as illustrated in Fig. 7B. Furthermore, an LOD of 38.05 nM is possible due to complexation of As^3+^ with lone pair electrons on the nitrogen components of three benzotriazole components, as shown in Fig. 7C, which subsequently causes an increase in the current signal at 0.68 V.^57^

Further use of rGO is exhibited by Tiwari *et al.*^58^ where GO was electrochemically reduced and combined with electrodeposited gold to create an Au–rGO–SPE for detection of the arsenic-containing antibiotic, roxarsone. Methods for detection of roxarsone, or 4-hydroxy-3-nitrophenylarsonic acid, are of importance as it can accumulate in muscle tissue of poultry at high levels between 2 and 5 μg kg^−1^.^79^ By using a SWV method, an unmodified SPE was compared to the Au–rGO–SPE, alongside a rGO–SPE. When tested electrochemically, the modified SPE yielded an increase in peak current of 1.8 times for roxarsone concentrations in the range of 5–1000 μM in a phosphate buffer. Notably, the highest peak current was obtained in an electrolyte with a neutral pH of 7, with decreased peak currents observed in both acidic and alkaline phosphate buffers. In terms of reproducibility, after 42 days in storage at room temperature, the electrodes were tested towards a concentrated roxarsone solution, showing only a 2% drop in peak current compared to the original response. When applied towards detection of roxarsone in samples of chicken flesh extract, recoveries between 97% and 105.4% were recorded.

Comparing Au–rGO with other nanoparticle–rGO composites, Radinović *et al.*^80^ created a cobalt–rGO, and a cobalt–Au–rGO electode for voltammetric detection of As^3+^ in river and city supply water systems. In 1 M HCl at a deposition potential of −0.3 V and a short deposition time of 60 seconds, concentrations of As^3+^ were detected in the linear range of 30–1000 μM with a low LOD of 3.06 μM. Notably, the presence of common tap water contaminants such as Cu^2+^, did not affect the detection of arsenic, indicating good selectivity. For real world river and city water samples, a defined peak was observed at 0.13 V, with an intensity of 1.15 mA cm^−2^, although no LODs are reported.

**Fig. 8:**
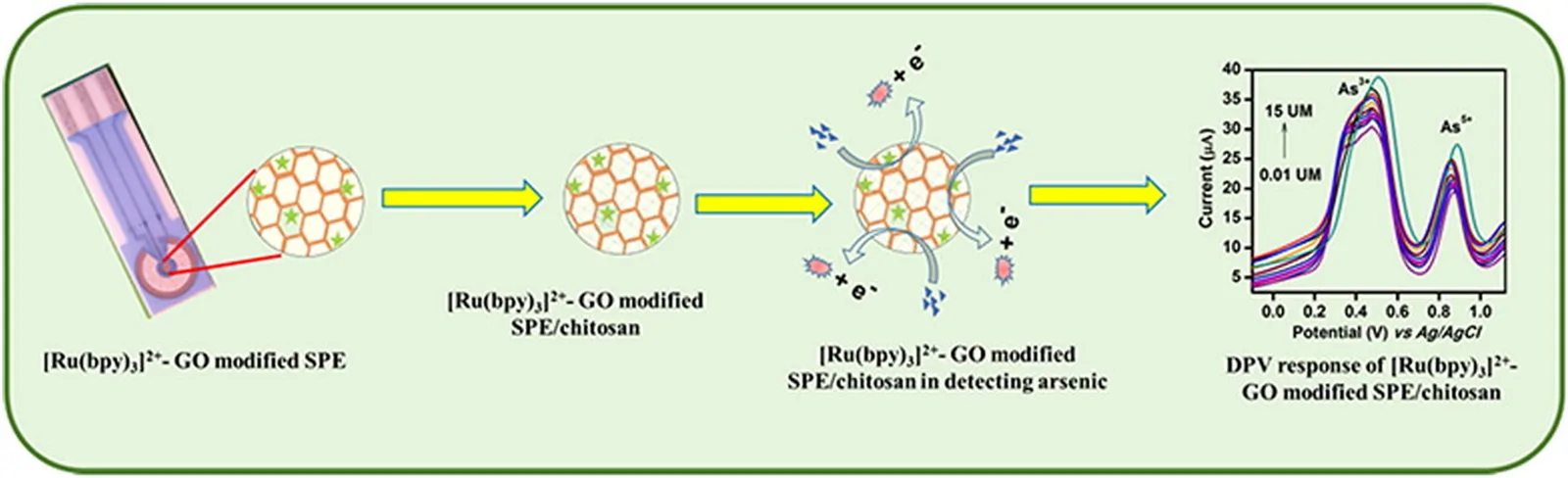
Schematic showing the fabrication process of the [Ru(bpy)_3_]^2+^–graphene oxide SPE and its mechanism towards detection of arsenic using a differential pulse voltammetry method. Figure reproduced from ref. 56 with permission from Elsevier,^56^ copyright 2018.

Many of the detection systems available for As, focus primarily on the As^3+^ species as As^5+^ is routinely more difficult to quantify with voltametric methods. To facilitate the detection of As^5+^ions without the need for highly acidic electrolytes, Gumpu and coworkers employed a SPE modified with ruthenium bipyridine graphene oxide ([Ru(bpy)_3_]^2+^–GO) with high surface area to facilitate detection of total As in water. The electrode fabrication process is outlined in Fig. 8.^56^ The authors first created a mixture of 10 mg mL^−1^ [Ru(bpy)_3_]^2+^–GO and chitosan, which was then drop-cast onto the working electrode of a SPE in a 2 μL volume. This modification increases the electroactive area from 18.3 mm^2^ to 41.6 mm^2^.

During an interference study with other metal ions, only Hg^2+^ presented an interference that decreased the current signal and peak potential for As^5+^, however, this only occurred when the Hg^2+^ concentration became 20 times higher than that of the As concentration.

It is clear that the functional groups and the enhanced electronic structure and active sites of rGO modified electrodes allow for ease of attachment for fabricating multi-faceted surface modifications and these factors have been shown above to result in promising avenues for research.

### 5.2. Polymers

Modifications are often paired with conducting polymers such as polyaniline as precoating layer due to its high stability and relatively low cost.^81^ Hamid Kargari and coworkers^82^ employed a sensor based on the combining of polyaniline with the cationic polymer, poly(diallyldimethylammonium chloride) alongside GO nanosheets. The acrylic acid functionalised GO nanosheets provided a large surface area and when combined with the cationic poly(diallyldimethylammonium chloride), facilitates a higher affinity for As^3+^ due to the electrostatic interactions between the surface and target ion species. The sensor achieved a 0.12 μM LOD but this particular study relies on a traditional glassy carbon electrode within a three-electrode setup rather than a SPE and therefore will have limitations when translating that into the field.

### 5.3. Covalent organic frameworks

**Fig. 9:**
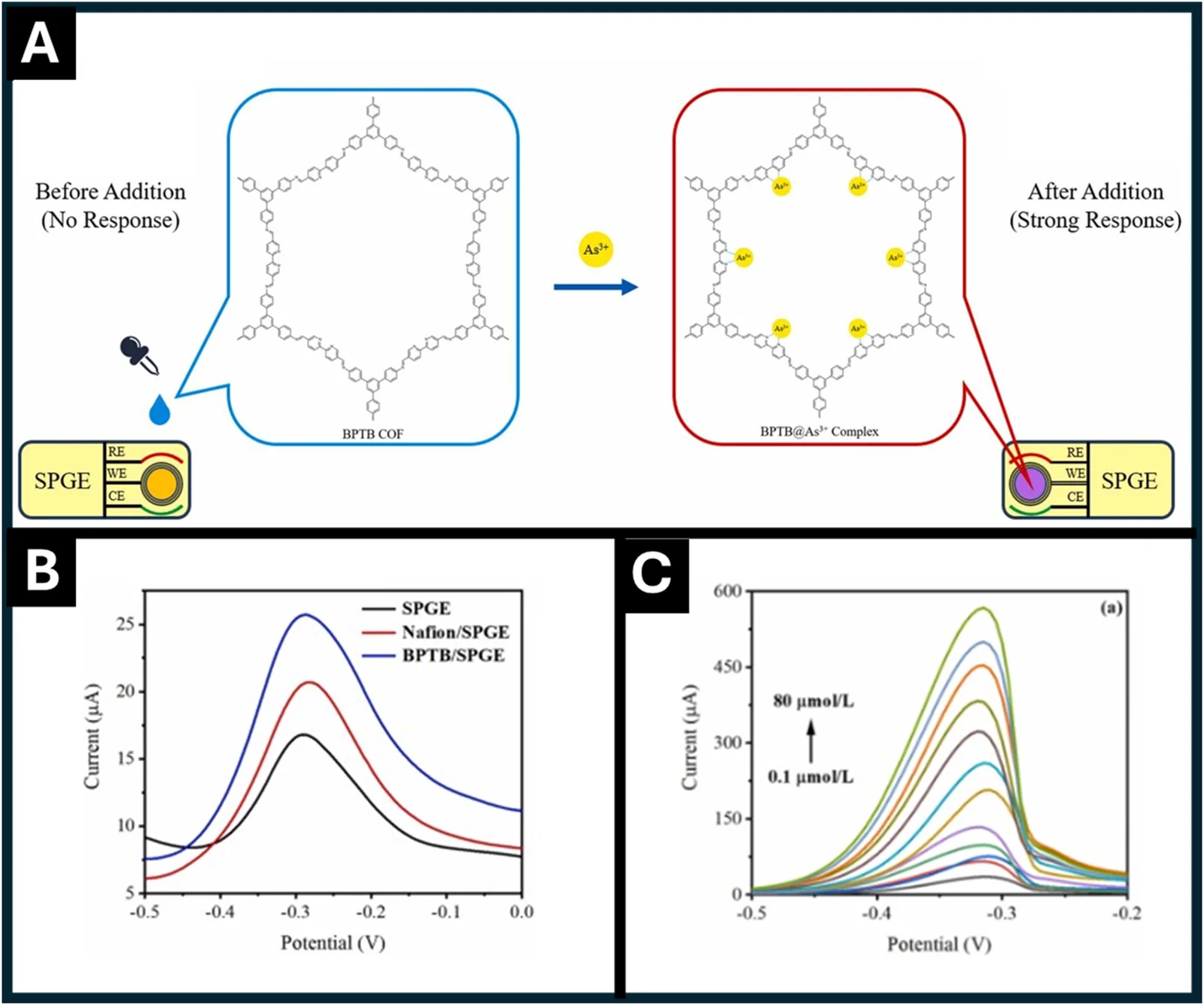
A) a depiction of the process for As^3+^ ion interaction with the bipyridine-functionalised covalent organic framework. B) Square wave anodic stripping voltammetry on unmodified and modified SPEs in 0.1 M acetate buffer at As^3+^ concentrations at 20 μmol L^−1^. The method used an optimised deposition potential of −1.4 V and a deposition time of 120 seconds. C) Square wave anodic stripping voltammetry showing the electrochemical response of 60 μmol L^−1^ As^3+^ concentrations in the 0.1–80 μmol L^−1^ with a deposition potential of −1.0 V and a 300 second deposition time. Figure reproduced from ref. 83 with permission from Elsevier,^83^ copyright 2024.

Liu and coworkers present a novel approach to As^3+^ determination by designing a covalent organic framework (COF) as an SPE modification.^62^ The bipyridine-containing COF with a large surface area is depicted in Fig. 9A. COFs have a unique ability to host metal ions for sensing due to their regular pore structure and large surface area. To create the bipyridine COF, a bipyridine and 1,3,5-tris-(3-animophenyl)-benzene in tetrahydrofuran solution was created. Once dissolved, acetic acid was added to the mixture and left at room temperature for one week. After subsequent high speed centrifugation, the bipyridine COF was extracted in the form of a yellow solid, mixed with a Nafion/ethanol dispersion and dropped onto the working electrode surface. Characterisation by XRD, FTIR, SEM and TGA confirmed the expected COF structure, with a uniform, porous surface structure. Using XPS analysis, the authors confirmed that the bipyridine sites of the COF have a high affinity for As^3+^, which intensifies the current during SWASV analysis (Fig. 9B and C), leading to an ultralow LOD of 38.09 nmol L^−1^.

Although graphene, polymer, and COF-modified carbon electrodes have each demonstrated notable improvements in arsenic sensing performance, all approaches face limitations from a material and/or fabrication perspective. Graphene and rGO offer excellent conductivity and surface areas, but suffer from aggregation, defect variability, and low selectivity toward arsenic species, with additional issues of fouling and oxidative degradation during prolonged operation. These limitations may be mitigated by introducing heteroatom dopants or functional groups to improve chemical affinity, through incorporating metal nanoparticles or polymeric spacers to prevent sheet restacking, and by employing scalable fabrication techniques (such as inkjet or laser-assisted printing for uniform electrode coatings). Polymer-based modifications, while easy to process and cost-effective, face problems of oxidative instability, swelling, and inconsistent film morphology reducing reproducibility. These issues can be addressed through crosslinked or copolymer designs with improved chemical robustness, hybridisation with graphene or metal nanoparticles to enhance conductivity, and the use of molecular imprinting or biomolecular dopants for greater selectivity. COF-modified electrodes provide high porosity and tuneable binding chemistry but are limited by poor conductivity, complex synthesis, and hydrolytic degradation. Advances in conductive COF–carbon composites, incorporation of stable linkages such as β-ketoenamine or triazine, and improved deposition methods like *in situ* growth or electrophoretic coating could overcome these barriers. Continued optimisation and validation of these materials under real environmental conditions will be vital for translating laboratory-scale devices into reliable, field-ready arsenic sensors.

## 6. Biological modification

A biosensor is defined as a device that harnesses the reactions of biological materials such as enzymes, proteins, and antibodies to detect a range of analytical targets using electrical signals.^84^ Biosensors have many advantages in As detection applications, owing to their versatility, robustness and the unique selectivity afforded by the biological recognition elements they employ, allowing for the differentiation of distinct As species.^85^

Biosensors can be broadly classified into two main categories based on the type of biological component they use. The first category includes enzyme-based biosensors, which leverage the catalytic activity of specific enzymes to detect arsenic through redox reactions or the inhibition of enzymatic activity. This approach offers exceptional selectivity due to the enzyme's ability to recognise specific chemical structures. The second category encompasses DNA-based biosensors, which use nucleic acid sequences, such as aptamers or oligonucleotides, that can selectively bind to arsenic ions. These DNA-based sensors are highly sensitive and can be engineered to target specific arsenic species, making them effective even at trace levels.

### 6.1. Enzyme-based biosensors

One example of an enzyme used in sensors is acetylcholinesterase (AchE). AchE is predominantly found in muscles and nerves for the purposes of hydrolysing acetylcholine into acetic acid and choline, finding uses in medical treatments for Alzheimer's disease.^86^ A dual biosensor with AchE and acid phosphatase cross-linked onto a carbon SPE revealed the detection of both prominent species, As^3+^ and As^5+^ in concentrations as low as 2.0 and 35.9 μM, respectively, in water samples.^87^ Analysis of both species was attempted in wine samples, however, only As^5+^ could be detected with a satisfactory level of recovery, relative standard deviation, and confidence interval for mean.

Additionally, a disposable AchE-based sensor based on a 4-acetoxyphenol substrate was reported by Li *et al.*,^85^ showing a simplified sensor fabrication route due to the electrochemical properties of the hydrolysis product, hydroquinone, mitigating the need for an external redox mediator.^88^ The sensor harnesses the pseudo-irreversible inhibition mechanism of AchE by As^3+^, enabling direct amperometric detection on a carbon SPE. This sensor demonstrated a broad linear range of 2–500 μM paired with an LOD of 2.2 μM. Notably, the sensor maintained operational stability for up to 150 days at ambient temperature. Similarly, other work by Sanllorente-Méndez *et al.*^89^ reported a sensor modified with a covalently bonded AchE on the surface. In optimised conditions in a Britton–Robinson buffer at pH 7, the biosensor achieved a 1.1 nM LOD in the linear range of 1–10 nM with RSDs below 4% for both repeatability and reproducibility. Although, interference studies identified Hg^2+^ as a significant interference, alongside minor interference from Ni^2+^ and Cu^2+^ at higher concentrations. Application to spiked tap water confirmed the method's applicability to real world samples.

In further developments of enzyme-based screen-printed biosensors, Núñez *et al.*^90^ demonstrated the use of *Alcaligenes faecalis*, a bacterium containing the arsenite oxidase enzyme immobilised onto a AuNP-modified SPE for detection of As^3+^. The biosensor exploits the ability of the enzyme to oxidise As^3+^ to As^5+^, which generates a measurable electrochemical signal. Two optimised detection methods were established, revealing a LOD of 6.61 μM at pH 7 and an applied voltage of 300 mV, whereas a 1.84 μM LOD was achieved at pH 12 and 700 mV. Despite modest sensitivity compared to similar sensors, the electrode showed good selectivity again common metal interferences and maintained stable for up to six days and 15 measurements. Application to As-contaminated river water samples yielded results in good agreement with ICP-MS analysis, highlighting the potential of biosensors for field-deployable As monitoring.

### 6.2. DNA-based biosensors

**Fig. 10:**
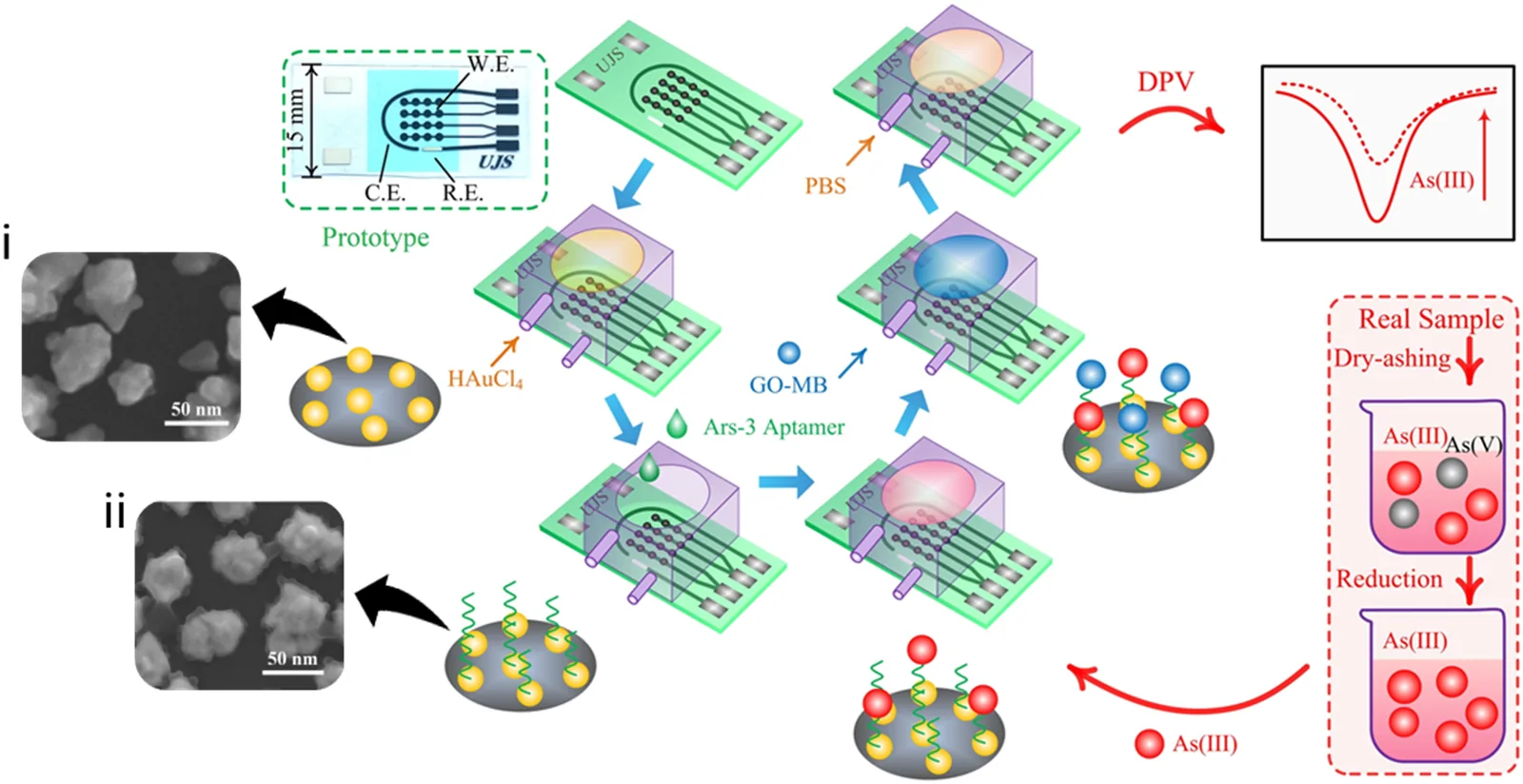
Principles of the Ars-3/AuNPs/SPE with the enhanced signal of graphene oxide–methylene blue. The insets reveal SEM images of i) gold nanoparticles deposited onto the SPE surface and ii) the Ars-3 gold nanoparticle modified working electrode surface. Adapted from ref. 92 with permission from Elsevier,^92^ copyright 2021.

Aptamers, a type of single-stranded DNA or RNA, are commonly utilised in sensing as they bind specific target molecules. Using the traditional 3-electrode electrochemical set up, Vega-Figueroa *et al.*^91^ modified a gold substrate with an arsenic-specific aptamer to detect As^3+^ concentrations in the linear range of 0.05–10 mg L^−1^ (using an electrochemical impedance method). Building on this research, Zhang and coworkers developed a SPE with electrodeposited AuNPs in combination with a self-assembled As-specific aptamer layer.^92^ The resulting sensor was then incubated with a graphene-oxide and methylene blue nanocomposite for 20 minutes. Notably, the SPE's working electrode component was comprised of a 16-microelectrode array, each of 1 mm *Ø*. The design and operating principle of this system are summarised in Fig. 10, which illustrates the stepwise modification of the SPE surface, from AuNP electrodeposition to aptamer immobilisation and GO–MB assembly, as well as the detection process based on a DPV method. The figure inset i) shows the morphology of the gold nanoparticles on the electrode surface, alongside the inset ii) which shows the morphology changes when the As-specific aptamer is included. The figure also shows how As^3+^ binding induces conformational changes within the aptamer layer, leading to measurable current variations. Opposed to assessing arsenic levels in water samples, this worked explored contamination in shellfish. The measurable samples were obtained *via* a dry-ashing method, whereby the edible tissue was fired in a high temperature furnace to obtain an ash, which was then dissolved in nitric acid to obtain an analysis-ready sample. The LOD was calculated to be 2 μg L^−1^.

Enzyme and DNA-based biosensors represent two complementary approaches for electrochemical arsenic detection. Enzyme-based systems, employing biorecognition elements such as acetylcholinesterase, acid phosphatase, and arsenite oxidase, demonstrate dependable As^3+^/As^5+^ speciation with good reproducibility and long-term stability of up to approximately 150 days. In contrast, DNA aptamer platforms rely on target-induced conformational changes to achieve high molecular selectivity and sub-micromolar detection limits, even in complex sample matrices. Nevertheless, enzyme sensors remain prone to interference from coexisting metal ions and enzyme deactivation, while aptamer-based devices are constrained by immobilisation stability and incomplete validation of binding affinity. Whilst current research endeavours seek to mitigate such limitations, future progress should investigate hybrid sensor architectures that couple enzymatic signal amplification with aptamer specificity, integrating onto nanostructured electrodes with on-chip sample handling, and portable electrochemical readouts. Such developments would enable the transition of arsenic biosensors from laboratory demonstrations toward robust, field-deployable platforms, for real-time environmental monitoring.

## 7. Conclusion

Detecting arsenic in drinking water remains a global challenge, especially in regions where long-term exposure exceeds WHO limits. While laboratory-based techniques like ICP-OES and ICP-MS offer high accuracy, they are costly, labour-intensive, and unsuitable for routine, widespread monitoring. This highlights the need for portable, low-cost, and user-friendly alternatives for rapid, on-site analysis.

**Fig. 11:**
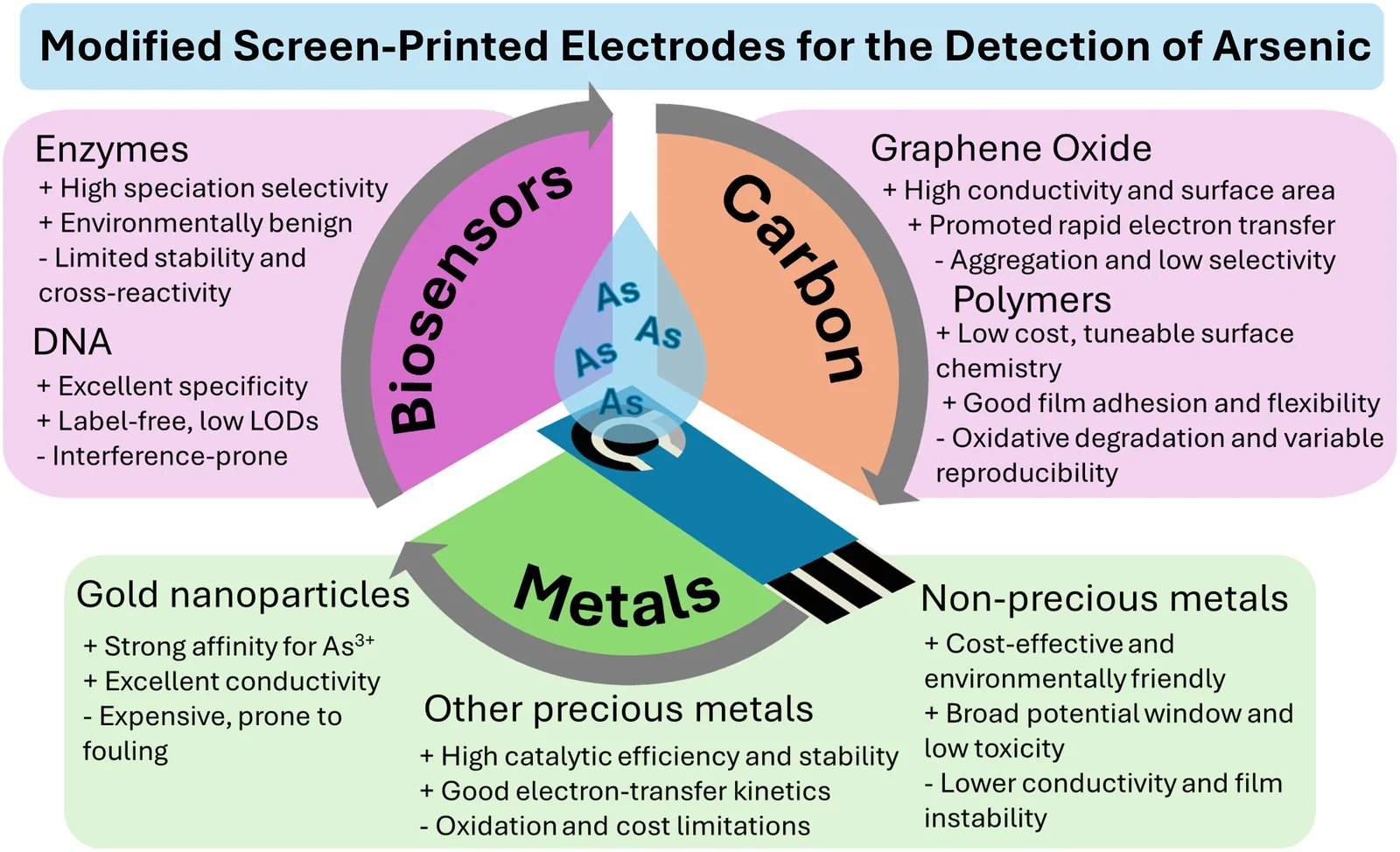
A summary of the electrode modifications explored within this review. The advantages and disadvantages of using biosensor, carbon-based, and metal-based modifications are briefly summated.

SPEs offer a promising solution due to their scalability, versatility, and ease of use. Beyond the flexible design of SPEs themselves, performance has been significantly enhanced through modifications with nanomaterials (*e.g.*, gold nanoparticles, carbon nanotubes), conducting polymers, and surface functionalisation. These strategies, summarised in Fig. 11, improve sensitivity, selectivity, and detection limits, enabling trace arsenic detection in line with regulatory standards.

However, practical concerns remain. The use of precious metals or rare materials raises sustainability and cost issues, potentially limiting large-scale deployment. Additionally, complex fabrication processes can hinder accessibility, especially in resource-limited settings. Simplified, scalable modification techniques are needed to overcome these barriers. SPEs enable real-time monitoring in both urban and remote areas, where conventional lab methods are impractical. Crucially, future studies utilising SPEs towards arsenic detection should not aim to further lower the currently capable limits of detection, as they are already well below the WHO and EPA recommended limits. Instead, SPE modifications should be designed to effectively perform within a wide variety of environmental matrices (namely: water samples) as the accuracy and reproducibility of the observed electrochemical signal output is the key challenge to the wide-spread implementation of this technology and one that must be overcome before it can reach commercial maturity.

Incorporating machine-learning algorithms into electrochemical data analysis could greatly enhance selectivity and reliability by distinguishing overlapping redox signals, compensating for environmental drift, and enabling adaptive sensor calibration in the field. Coupled with microfluidic handling and *in situ* spectroelectrochemical monitoring, such intelligent systems could evolve into autonomous, networked sensors capable of real-time arsenic surveillance across diverse environmental settings.

Ongoing research into novel materials, simplified fabrication, and surface stability—while reducing reliance on costly catalysts—continues to expand the potential of SPEs. As a transformative technology, SPEs can greatly enhance global public health by enabling frequent, reliable, and accessible arsenic monitoring.

## Conflicts of interest

The authors declare no competing interests.

## Data Availability

The authors declare that they will provide all resources/data utilised to produce this review article if they are requested.
